# Reducing Waste in Healthcare through Occupational Safety and Health Measures: A Study of Manufacturing Industries in Taiwan

**DOI:** 10.3390/healthcare9111476

**Published:** 2021-10-30

**Authors:** Ya-huei Wang, Cheng-Ming Chang, Hung-Chang Liao

**Affiliations:** 1 Department of Applied Foreign Languages, Chung Shan Medical University, Taichung 402306, Taiwan; yhuei@csmu.edu.tw; 2 Department of Medical Education, Chung Shan Medical University Hospital, Taichung 402306, Taiwan; 3 Institute of Labor, Occupational Safety and Health, Ministry of Labor, New Taipei City 221004, Taiwan; ming@mail.ilosh.gov.tw; 4 Department of Health Policy and Management, Chung Shan Medical University, Taichung 402306, Taiwan; 5 Department of Medical Management, Chung Shan Medical University Hospital, Taichung 402306, Taiwan

**Keywords:** occupational safety and health (OSH) management, education/training, work permission, hazard assessment, personal protective equipment

## Abstract

Occupational accidents and diseases cause the loss of valuable workers and lead to high healthcare expenses. Because occupational accidents and diseases are ascribed to inadequate working conditions and work environments, they can be prevented through a well-established occupational safety and health management system, which can ensure workers’ health and reduce the expense of healthcare. The study investigated the shortage of work-related occupational safety and health (OSH) measures in medium-sized manufacturing industries. This study mainly focused on qualitative interviews with 15 labor inspectors and 25 business executives from OSH participating to investigate the problems of occupational safety and health in the manufacturing industries in Taiwan. The results of a qualitative study show that the most important problems with OSH management are employers’ negligence and workers’ insufficient knowledge about OSH management. The research results revealed the following eighteen significant shortcomings of OSH management: employers care mostly about production profit and do not care much for OSH; OSH data collection and OSH planning are not suitable for the workplace; many managers of OSH affairs are not qualified, in terms of their professional or academic backgrounds; and the repair of workplaces’ roofs often results in falling accidents, especially before or after a typhoon, because of workers’ failure to use safety belts and/or to follow OSH guidelines. In order to address the shortcomings and bottlenecks, the study also presented recommendations for how to implement and revise the OSH Act and how to research and enhance OSH management. The results of this study will not only supply the Ministry of Labor (Taiwan) with data to plan the strategy of OSH management but also will allow employers and workers to improve OSH management in the workplace in order to prevent the occurrence of occupational accidents.

## 1. Introduction

Occupational accidents may cause the loss of valuable workers and high healthcare expense [1]. Those suffering from occupational hazard may be afflicted with occupational diseases, work-related illness, or loss of life [2,3]. Because occupational accidents and diseases are ascribed to inadequate working conditions and work environment, they can be preventable under a well-established occupational safety and health management system, which serves as a quality management technique to ensure workers’ health and reduce the expense of healthcare [4,5]. Hence, it is vital for companies to promote health and safety in the workplace because health and safety have a considerable influence on the reputation of a company. Most Taiwanese manufacturing contractors’ attitudes toward occupational safety and health (OSH) work generally meet only the minimum requirements of the OSH Act. In addition, the competition in such a saturated environment is fierce, and the profit of the manufacturing industry is often reduced. Rose et al. [6] and Tompa et al. [7] assumed that the cost of OSH affects financial performance. In order to survive, medium-sized manufacturing industries often do not invest much in safe facilities. This situation is similar to that of medium-sized manufacturing industries in other countries and results in many more hazardous risks for medium-sized manufacturing industries than other industries [8].

In the past, manufacturing industries mainly focused on the production system, and their main purpose was the pursuit of profits. These industries did not pay much attention to the importance of overall OSH management, which caused many occupational disasters [9,10]. Moreover, the OSH management system is not perfect in manufacturing industries. Hence, the risks of accidents for workers have increased [11]. These accidents are more likely to cause many workers’ families financial and social problems. Serious social burdens may also lead to permanent disabilities, deaths, and/or economic losses [12].

According to 2019 Ministry of Labor (Taiwan) statistics [13], between 2005 and 2017, 473,758 workers or their families applied for labor insurance benefits with the Taiwanese government, and 3887 of these workers died from occupational injuries. Thirty-four thousand, one hundred and six people have been disabled; that is, more than 145 workers (145.77) face the danger of workplace accidents every day, and more than 1 (1.20) worker dies each day, on average. Moreover, there are 187,833 workers in in the manufacturing sector who applied for occupational injury insurance, accounting for about 40% (39.65%) of the claims for total occupational injury insurance among workers. The number of deaths and disabilities caused by occupational injuries in the manufacturing industries in Taiwan are much more than the number in more advanced countries. In the United Kingdom, approximately 20 workers per year are killed by occupational injuries in manufacturing industries, and approximately 600 workers are disabled [14]. In addition, occupational injuries will cause companies legal expenses and the loss of valuable workers. These findings clearly indicate that occupational accidents and diseases are closely related to OSH management and must be properly addressed by industries [15].

Injuries and fatal accidents connected with OSH frequently occur. France, Italy, and Spain reported statistics on fatal accidents regarding OSH, with 2.57, 2.42, and 2.3 fatal accidents at work per 100,000 workers employed in 2015, respectively [16]. In Great Britain, between April 2019 and March 2020, 111 workers were killed because of fatal occupational injuries, and 92 people were fatally injured due to work-related activities [17]. In Pakistan, the reported occupational deaths and injuries are greater than in other developing economies. Annually, Pakistan has approximately 7444 fatal accidents and 5,680,740 occupational accidents that prevent workers from going to work at least for three days [18]. Besides, OSH is incomplete enough to make workplaces dangerous in certain countries. For example, in Africa, most OSH regulations are obsolete. There is insufficient implementation and low reported data on accidents and occupational disasters [19]. Stewart and Nite [20] even found that black mine workers did not have remedial mechanisms for either individual or collective actions. In Greece, 100 cases of occupational accidents or related occupational disasters from the 25,000 victims were reported to have died each year [21]. Based on these studies, safety is a primary concern.

A large number of occupational exposures and activities are associated with an increased risk of occupational diseases, injuries, or fatalities, including occupational cancer (such as lung cancer and prostate cancer), respiratory disease (such as asthma and pneumoconioses), and musculoskeletal conditions (such as limb disorders and low back pain) [22]. However, these occupational injuries, diseases, or even fatalities can be avoided under a well-established OSH management system [4,5]. After an occupational accident occurs, it will not only affect the worker but also cause a burden for the worker’s family. The cost to society is high. If an accident is irreversible, there will be detrimental impacts on people and the environment.

OSH is affected by numerous factors, including hazards in the working environment, social factors, individual behaviors, and access to health services [23]. In recent years, researchers have proposed many well-known OSH methods and strategies. Corbell [24] declared that professional safety certification has become the current trend. Consequently, OSH professionals must have considerable understanding of work environments and equipment as well as sector-specific skills. Hee et al. [25] pointed out that more than 80% of occupational accidents were caused by human and organizational factors. Thus, when the cause of an accident is not investigated, and such organizational factors exist, occupational injuries, diseases, and fatalities will occur eventually, which not only increases the cost in economics but also the cost in medical and health care, hence causing unnecessary burden and waste in the medical and health care system.

In order to address the flaws of the current OSH management system in medium-sized manufacturing industries, the study intended to take an investigation in the shortcomings of the system and hence set up a well-establish OSH management system to provide a secure work conditions and work environment for labors. This study mainly focused on qualitative interviews with 15 labor inspectors and 25 business executives from OSH. The specific aims of this study are:

- To understand the status of the actual operations of OSH management;
- To understand the possible reasons for the poor performance of OSH;
- To understand the level of OSH management and the cause of the gaps; and
- To develop effective and feasible solutions for OSH’s deficiencies and provide recommendations for improvement of OSH management to authorities, enterprises, employers, and workers.

## 2. Methodology

The purpose of this study was to investigate the deficiencies in work-related occupational safety and health (OSH) measures in medium-sized manufacturing industries and further find solutions to OSH management in these industries. Because OSH is a complex system with complicated interactions with different disciplines [26,27], to achieve the above-mentioned goals, the study mainly focused on qualitative interviews with 15 labor inspectors and 25 business executives from OSH to investigate the problems of occupational safety and health in the manufacturing industries in Taiwan. The investigated enterprises had 50 to 99 workers. The qualitative research interviews could help researchers collect and analyze qualitative information in a systematic way and to delve deeply into the relevant issues [28,29]. From the qualitative interview results, the researchers collected and analyzed the deficiencies of OSH management. In addition, solutions and recommendations were provided for improvement to the OSH departments of medium-sized manufacturing industries.

The questions used in the qualitative interviews are listed as below:

- What are the major difficulties faced while promoting OSH? What are possible improvements or solutions?
- According to your experience, what is missing from and/or what are the bottlenecks in the management plan, occupational disaster-prevention plan, automatic inspection plan setting, and OSH education/training plan? What are possible improvements or solutions?
- Are there any flaws in working agreements or working guidelines? What are possible improvements or solutions?
- In your opinion, should dangerous items (such as gunpowder) be stored apart from one another to avoid accidental mixing? Is there any flaw in this system? What are possible improvements or solutions?
- According to your experience, what are the defects of the electricity prevention measures? What are possible improvements or solutions?
- According to your experience, what are the defects of hazard-assessment programs, occupational disaster-prevention plans, and hazardous workplace inspections? What are possible improvements or solutions?
- According to your experience, what are the defects in setting up OSH self-inspections plans? What are possible improvements or solutions?
- According to your experience, what are the defects in promoting the hazardous substance list, education/training, and labeling content of hazardous substances and harmful substances? What are possible improvements or solutions?
- According to your experience, is there any deficiency in the automatic alarm for flammable and high-pressure equipment? Is there any deficiency in assigning special personnel to handle hazardous materials manufacturing or disposal operations? What are possible improvements or solutions?
- According to your experience, what is the deficiency in the wearing and use of protective equipment? What are possible improvements or solutions?
- According to your experience, what are the defects in job-site monitoring, including items, frequency, the contents of the plans, measurement records, and follow-up improvement measures? What are possible improvements or solutions?
- According to your experience, what are the deficiencies in noise control; high-vibration work protection measures; protective measures for hot, cold, or wet indoor workplaces; and pits’ or storage operations’ ventilation and lighting? What are possible improvements or solutions?
- Based on your experience, what is the deficiency in workers’ health examinations? What are possible improvements or solutions?
- According to your experience, what are the deficiencies in hazardous mechanical equipment management? What are possible improvements or solutions?
- According to your experience, what are the defects in installing interlocking equipment in mechanical devices? What are possible improvements or solutions?
- According to your experience, what are defects in OSH education/training? What are possible improvements or solutions?
- According to your experience, what are the deficiencies in emergency-response plans? What are possible improvements or solutions?
- Do you have any other opinions on OSH in medium-sized manufacturing industries?

## 3. Results and Possible Solutions

Based on the qualitative interviews with 40 experts in OSH (15 labor inspectors and 25 business executives) regarding occupational safety and health in the manufacturing industries, the qualitative interview results and possible suggestions were made and shown as below.

### 3.1. What Are the Major Difficulties Faced While Promoting OSH? What Are Possible Improvements or Solutionss?

The major difficulties faced while promoting OSH is the indifference and lack of support from employers and workers’ misunderstanding of OSH. Many employers consider OSH to be a waste of money and a non-profit aspect of operations. For most medium-sized manufacturing industries, OSH affairs are considered subsidiary work, work that is required in order to comply with the OSH Act. For manufacturing companies with fewer than 100 employees, the Act requires business executives only to address the issues. There is no need to set up OSH departments. Hence, many enterprises send only general affairs personnel to receive OSH education/training. These personnel are usually busy with their main business affairs and cannot focus on OSH work. In order to address the indifference and lack of support from employers, companies should assign managers with engineering backgrounds instead of general affairs personnel to receive OSH education/training.

Workers’ misunderstanding of OSH can be discussed both in terms of formal education and on-the-job training. In terms of formal education, for the medium-sized manufacturing sector, workers’ educational backgrounds can generally be divided into junior high school, senior high school, and college. In the past decades, many workers graduated only from junior high school and had very little training in OSH. Today, workers who graduate from vocational high schools often encounter more hazardous work situations. Teachers have also reminded such workers of safety precautions. Moreover, these workers have more ideas about “safety” in the factory. However, having not received formal OSH education, these workers still lack the overall concept of OSH. Workers who graduated from junior college, because of their high level of knowledge, pay more attention to their rights. They receive formal OSH education during their skill development. They also pay more attention to safety in the workplace and are more willing to accept the benefits of OSH.

In terms of on-the-job training, a factory can hold its own OSH training. However, companies commonly face some difficulties in training newly hired workers in OSH because of the lack of OSH lecturers and appropriate teaching materials. Most on-the-job training only has the initial effect of reminding workers to pay attention to OSH. However, it is not easy to verify the success of OSH.

To address workers’ misunderstandings of OSH, junior high schools and vocational high schools should teach OSH courses in order to enhance students’ perceptions of OSH. Also, The OSH Act should have a system for auditing OSH education/training. With an internal auditing system, workers have more chances to master OSH knowledge. An external audit system would enable authorities to understand the implementation of education/training. In addition, internship teaching courses can be added to the education/training courses. When trainees return to their companies for self-education/training, the education/training content can be easily taught. Moreover, a stricter auditing system should be established for licenses in education/training institutions.

### 3.2. According to Your Experience, What Is Missing from and/or What Are the Bottlenecks in the Management Plan, Occupational Disaster-Prevention Plan, Automatic Inspection Plan Setting, and OSH Education/Training Plan? What Are Possible Improvements or Solutions?

Companies often do not know how to draw up OSH plans. Many companies plagiarize and do not examine the applicability of the copied information. In addition, most of the OSH personnel in medium-sized manufacturing industries often work in general affairs or are accountants or operators. Due to these employees’ limited knowledge, they do not understand the performance of the machines and equipment in their factories. Setting OSH standards is thus difficult for them. Some enterprises ask OSH consultants to set OSH standards. However, some enterprises consider only commercial profits and ignore business ethics, so OSH standards are omitted. Hence, it is doubtful that these enterprises will correct OSH performance.

To overcome the bottlenecks in the management plan, occupational disaster-prevention plan, automatic inspection plan setting, and OSH education/training plan, enterprises should assign a senior manager the responsibility for OSH or establish an OSH department that will contribute to the implementation of OSH. Additionally, personnel who are actually engaged in OSH must have engineering backgrounds, be familiar with the OSH content, and know how to control the OSH procedure. In addition, industrial authorities should establish OSH counseling organizations, such as non-profit organizations. The authorities should subsidize these organizations to assist manufacturers in setting OSH standards.

### 3.3. Are There Any Flaws in Working Agreements or Working Guidelines? What Are Possible Improvements or Solutions?

Some enterprises have working agreements concerning OSH and require outsourced workers repair roofs and water and electrical pipelines after typhoons. These agreements subject workers to the risk of falling while repairing wires, roofs, and so on. Most medium-sized manufacturing industries ignore the hazards of falling and being electrocuted. In addition, such working agreements may also involve some smaller or downstream enterprises or outsourcing industries to complete these tasks. These smaller or downstream enterprises or outsourcing industries may not know how to set up working agreements to protect workers’ OSH. The effectiveness of policies for preventing fires is also poor. In particular, production or maintenance departments sometimes use electric welding equipment to repair equipment and facilities. However, these departments usually do not notify OSH management personnel that such electric welding equipment may cause fires. Some on-site manufacturing personnel do not know that they must apply for fire permits in advance.

To address the flaws in working agreements or working guidelines, from the point of view of loss control, if OSH performance is quantified as a quantified production performance, an employer should more easily accept the OSH concept. If OSH can be integrated into an organization’s performance assessment system, the employers should be more willing to accept OSH. The formulation and implementation of an OSH performance appraisal system will thus be significantly more valuable. In addition, it is necessary to strengthen the awareness of possible accidents caused by roof repairs during the yearly typhoon season. Organizations should also strengthen OSH management with a working agreement with contractors. In addition, work permission for preventing fires depends on the promotion of education/training. Enterprises should continuously promote autonomous management. Additionally, the penalty for major work safety incidents should be increased in order to prevent fires.

### 3.4. In Your Opinion, Should Dangerous Items (Such as Gunpowder) Be Stored Apart from one Another to Avoid Accidental Mixing? Is There Any Flaw in this System? What Are Possible Improvements or Solutions?

There are currently very few separate storage areas for dangerous materials. Most dangerous materials are stored in piles in the same warehouse. Most companies do not properly plan for the storage of dangerous materials during plant design. The OSH Act stipulates only that dangerous substances must be stored separately, and there are no specific segregation provisions. To address the flaw in this system, OSH education/training courses should strengthen the awareness of possible accidents caused by dangerous materials. If an organization has a shortage of storage facilities, dangerous materials can be stored in a partitioned area to prevent pollution or explosion.

### 3.5. According to Your Experience, What Are the Defects of the Electricity Prevention Measures? What Are Possible Improvements or Solutions?

The most common occupational hazard is touching a charged body. Many workplace devices do not comply with electrical regulations. Especially in the fields of hardware manufacturing, grinding, and humidification systems, these devices usually do not have ground wires or leakage circuit breakers. OSH managers should pay special attention to the possibility of explosions caused by electrical sparks. Especially in medium-sized manufacturing, most electrical equipment does not have explosion-proof measures. Additionally, fire departments inspect fire-protection facilities. However, the explosion-proof measure of the fire law and the OSH Act are not the same, resulting in two sets of standards and confused factories. Hence, a comprehensive electrical disaster inspection has been conducted. The inspection of electrical boxes is also a focus for preventing electrical shock. However, many employers do not know the importance of electrical box installations, and workers do not know how to look after their own safety. They lack a good understanding of how to prevent electrical shock. Because industries care only about cost, they may ignore the faults in wiring installations of electrical facilities and hence cause injuries to workers. To prevent being electrically shocked, workers should examine electrical equipment to avoid the occurrence of occupational disasters. Many factories have a large demand for electrical equipment. Even neglecting safety inspections of leakage circuit breakers often causes disasters.

In order to address the defects of the electricity prevention measures, safety education/training for electricians should be improved. If electrical equipment is not properly installed, the electrician should bear legal responsibility. The Taiwan Power Company shall stop supplying power, following the guidelines of electrical specifications, to factories that do not install electrical equipment as required. Manufacturing factories entrust the installation of their electrical equipment and maintenance to electrical and mechanical engineering companies. Strengthening and outsourcing the responsibility of suppliers for the dangers of electric shock accidents could also solve this problem.

### 3.6. According to Your Experience, What Are the Defects of Hazard-Assessment Programs, Occupational Disaster-Prevention Plans, and Hazardous Workplace Inspections? What Are Possible Improvements or Solutions?

The implementation of hazardous workplace reviews and inspection methods will contribute to the implementation of facility safety. However, the effectiveness of these procedures is not obvious because cost is still an employer’s biggest consideration. Medium-sized manufacturing industries have hardly implemented hazard risk-assessment and occupational disaster-prevention plans because such plans involve risk-assessment tools and assessment methods. These organizations do not know how to use these tools well. Hence, in order to address the defects of hazard-assessment programs, occupational disaster-prevention plans, and hazardous workplace inspections, OSH education/training courses should strengthen the formulation of plans to facilitate practical application in the implementation of OSH. In addition, industry authorities should prepare a set of simple OSH assessment manuals for factories to understand how to assess OSH risks.

### 3.7. According to Your Experience, What Is Missing from and/or What Are the Bottlenecks in the Management Plan, Occupational Disaster-Prevention Plan, Automatic Inspection Plan Setting, and OSH Education/Training Plan? What Are Possible Improvements or Solutions?

In terms of self-inspections of OSH, consulting companies provide most of the check-up tables for self-inspections in the factories. However, these tables contain all the items stipulated in the regulations rather than the factory’s needs. Some OSH managers do not know that they can and should design forms that suit their own factories. Instead, they use the check-up tables provided by consulting companies for their factories, hence failing to accurately monitor existing or potential hazards in their own factories. In order to overcome the bottlenecks in the management plan, occupational disaster-prevention plan, automatic inspection plan setting, and OSH education/training plan, inspectors must have professional backgrounds and inspection ability in order to strengthen the audit functions of the self-inspections by authorities.

### 3.8. According to Your Experience, What Are the Defects in Promoting the Hazardous Substance List, Education/Training, and Labeling Content of Hazardous Substances and Harmful Substances? What Are Possible Improvements or Solutions?

Labeling may serve only as a warning; however, most workers do not understand the contents. Therefore, in terms of education/training, the number of hours should be increased to increase workers’ awareness of dangerous materials, and enterprises should repeatedly remind workers in order to achieve OSH awareness. High schools should promote the recognition of hazards and harmful substances in the chemistry courses so that workers can receive early warnings about dangerous and harmful substances. In order to address the defects in promoting the hazardous substance list, education/training, and labeling content of hazardous substances and harmful substances, professional instructors with backgrounds in chemistry should lead workers’ education/training in chemical hazards. These professional instructors should also stress the importance of evaluating workers’ recognition of special chemical hazards. Moreover, chemistry textbooks should include the written description and graphic identification of dangerous and harmful substances to help workers identify dangerous and harmful substances.

### 3.9. According to Your Experience, Is There Any Deficiency in the Automatic Alarm for Flammable and High-Pressure Equipment? Is There Any Deficiency in Assigning Special Personnel to Handle Hazardous Materials Manufacturing or Disposal Operations? What Are Possible Improvements or Solutions?

The setting of automatic alarm equipment for flammable gas and high-pressure gas is generally good. Most factories employ full-time workers to manufacture or dispose of dangerous materials. However, authorities should check whether these workers have sufficient capacity to perform this job. In order to address these deficiencies, industrial authorities should set clear criteria for automatic alarm inspection and calibration, among which the explosion protection regulations for automatic alarms should be given priority. Besides, OSH lecturers on the manufacturing and disposal of hazardous materials should receive regular training.

### 3.10. According to Your Experience, What Is the Deficiency in the Wearing and Use of Protective Equipment? What Are Possible Improvements or Solutions?

Most employees are not particularly willing to use personal protective gear. Some personal protective equipment imported from abroad may not be applicable, which greatly reduces the effectiveness of protective equipment. However, in order to increase laborers’ safety, while laborers are in training, industries should promote the importance of using personal protective gear and teach laborers how to use personal protective gear to decrease occupational disasters. Therefore, workers can develop the habit of wearing protective equipment. To address the deficiency in the wearing and use of protective equipment, lecturers should be educated on the use of personal protective equipment. Such education will cultivate the good habit in the next generation of workers of using personal protective equipment.

### 3.11. According to Your Experience, What Are the Defects in Job-Site Monitoring, including Items, Frequency, the Contents of the Plans, Measurement Records, and Follow-Up Improvement Measures? What Are Possible Improvements or Solutions?

With regard to the measurement and implementation of job-site monitoring to assess the risk to OSH, most organizations are concerned with the OSH measurement of the job site. However, most organizations do not know how to use the data derived from the measurement to improve OSH at job sites. After the OSH measurement is completed, there are no feedback measures at job sites—for example, for physical hazards, such as the checks of noise and vibration under OSA Act. If the occupational noise or vibration assessments are within occupational exposure limits in two consecutive years, and the relevant equipment is not changed at work sites, it is expected that the OSH measurement data can be reported to the competent authority to exempt the following year’s inspection. The examination of chemical substances can prevent emergency hazards and occupational diseases. One measurement every six months can prevent occupational diseases. In addition, the use of direct-reading instruments is effective for preventing emergency hazards, such as gases and aerosols. To prevent organizations from providing false results of job-site monitoring and effectively improving their working environments, sampling teams should conduct sampling inspections.

To address the defects in job-site monitoring, including items, frequency, the contents of the plans, measurement records, and follow-up improvement measures, organizations should conduct further statistical analysis of the data obtained during the measurement of job-site monitoring to learn more, including information about existing or potential hazards at job sites. In addition, risk assessments, detailed preventive measures, and follow-up assessments should be used. This information will help companies understand the hazards of their working environments, help prevent occupational diseases, and avoid emergency hazards. In addition, sampling tests of job-site monitoring should be strengthened in order to understand the difference between the records and the results of the sampling test.

### 3.12. According to Your Experience, What Are the Deficiencies in Noise Control; High-Vibration Work Protection Measures; Protective Measures for Hot, Cold, or Wet Indoor Workplaces; and Pits’ or Storage Operations’ Ventilation and Lighting? What Are Possible Improvements or Solutions?

Noise is a common problem in the manufacturing industry. Most medium-sized manufacturing industries are very old and have problems pertaining to the equipment itself or lack of space. Noise control is often ineffective, and it is also the source of the most complaints. Other problems, such as high vibrations, are rare. To address these deficiencies, the engineering technology involved in noise improvement is very professional in nature. It can help if academic organizations assist in improving performance. In addition, one way to control noise is if the authorities subsidize the elimination of unsuitable equipment.

### 3.13. Based on Your Experience, What Is the Deficiency in Workers’ Health Examinations? What Are Possible Improvements or Solutions?

Workers commonly have problems in their health examinations. OSH managers do not know how to inform workers about the relevant supporting measures to safeguard their health because most OSH managers lack professional medical knowledge. Additionally, because of competition in the market, some hospitals do not have good quality health examinations. Additionally, hospitals want workers to pay for additional health examinations. To address the deficiency, the authorities should urge employers to take relevant OSH measures based on the results of the workers’ health examinations to protect the health of the workers. Employers should establish labor health manuals. The contents should detail workers’ health examinations and suggest how to treat illnesses. When workers change jobs, they can bring their handbooks in order to continue tracking their health status.

### 3.14. According to Your Experience, What Are the Deficiencies in Hazardous Mechanical Equipment Management? What Are Possible Improvements or Solutions?

When considering the operations of cranes, most firms pay attention to the commander’s obtained licenses. In general, companies will send a worker to obtain a license only after inspection agencies have inspected the cranes. However, in practice, for the operators and commanders, most of the crane operations at work sites do not follow the operation guidelines because companies want only to save on operating costs. This lack of commanders frequently causes occupational disasters. To address the deficiencies in hazardous mechanical equipment management, the regulations governing the position of crane commanders in the firms should be strengthened, and administrative fines should be imposed to increase the safety recognition of firms using cranes.

### 3.15. According to Your Experience, What Are the Defects in Installing Interlocking Equipment in Mechanical Devices? What Are Possible Improvements or Solutions?

In some firms, in order to increase production, employers or workers dismantle or switch mechanical protection devices. This situation often results in the loss of the device’s protective function, causing injury to workers. To solve the defects in installing interlocking equipment in mechanical devices, the protection devices of mechanical devices should not be disassembled or switched. Additionally, authorities should encourage old machinery to be replaced in order to improve the safety of workers.

### 3.16. According to Your Experience, What Are Defects in OSH Education/Training? What Are Possible Improvements or Solutions?

Companies must plan for workers’ on-the-job training. The curriculum can combine production and safety, and companies can plan, execute, supervise, and evaluate the effectiveness of their education/training. OSH education/training is conducted mostly by the enterprises. If OSH executives lack specialization in the OSH Act, safety and health education/training will be ineffective. The source of education/training of lecturers is also an important issue. Due to the limited OSH academic departments in Taiwanese universities and colleges, there are not many professionals. Hence, it is important to cultivate the number education/training experts and lecturers. We should formulate a set of systems for the assessment of learning effectiveness. Moreover, if a certificate has an expiry date, OSH lecturers will be forced to continue updating their knowledge.

To address the defects in OSH education/training, the rules for education/training should include additional standard measures for the validity period of certificates and re-education/training so that OSH education/training can achieve lifelong learning. Only in this way can employers and workers be reminded of the importance of OSH. In addition, OSH should be introduced in elementary schools so that people can develop safe behavior and recognition from an early age. Moreover, organizations should establish an education/training manual for each laborer, and labor inspectors should check these manuals.

### 3.17. According to Your Experience, What Are the Deficiencies in Emergency-Response Plans? What Are Possible Improvements or Solutions?

Most companies can comply with the statutory provisions for the setting of emergency-response plans, but the degree of implementation varies. Many enterprises believe that emergency-response plans are just fire drills and that employers are subject to media propaganda. However, it seems that companies do not understand the difference between fire drills and the OSH emergency response. Companies do not know that many chemical disasters caused by fire disasters are OSH issues. Therefore, it is necessary to strengthen publicity and strengthen people’s understanding of OSH. To address deficiencies in emergency-response plans, authorities should target different kinds of manufacturing industries; they can assist companies in combining emergency-response plans and fire drills and offer to monitor the drills.

### 3.18. Do You Have Any Other Opinions on OSH in Medium-Sized Manufacturing Industries?

As for occupational disaster prevention for vocational students, some vocational schools have created cooperative education to assign students to do mechanical, electrical, or chemical work. Students are prone to occupational disasters when their concepts of OSH are not sufficient. Authorities must carry out special inspections in order to prevent occupational hazards with students. For those firms applying for cooperative education for occupational disaster prevention, the inspection of OSH shall be strengthened to prevent occupational disasters with students.

As for the advocacy for the prevention of occupational disasters, according to the interviewees, most workers’ understanding of OSH is insufficient, so the implementation of OSH is difficult. In order to strengthen workers’ attention to OSH, besides formal education, propaganda is also an important method. It is suggested that the advocacy for OSH can be effective for the prevention of occupational disasters. Advertising, broadcasting, labeling, or bulletin boards with simple text and pictures could be effective.

## 4. Discussion

While manufacturing industries take the pursuit of profit as the priority of organizational performance and management, they may overlook the OSH management system and hence lead to a decision bias regarding “functional stupidity” [30]. Montibeller and Winterfeldt [31] suggested that decision biases may come from motivational biases and cognitive biases, which may hence influence the level of OSH management. Cognitive bias refers to a systematic gap between the correct decision that decision makers should make and the actual decision they make for the task based on their own experiences and preferences [31]. Motivational biases, on the other hand, refer to a distortion in decision making in terms of their degree of desirability in events, outcomes, consequences, or choices [31]. While making an OSH decision, business executives or labor inspectors with functional stupidity may intend not to think and reflect on the OSH issues but consider OSH management in the direction of “economic profits,” thus resulting in occupational diseases, injuries, and even fatalities.

Hence, while establishing an OSH management system with reflexivity the relevant administrators and investigators should be cautious of their cognitive and motivational biases in order to set up a well-established OSH management system to minimize occupational injuries, diseases, or fatalities so as to increase the welfare of labors [32].

In order to eliminate the decision bias coming from certain labor inspectors or business executives, while investigating the OSH in medium-sized manufacturing industries, the researchers adopted qualitative research interviews with 15 labor inspectors and 25 business executives from OSH to collect and analyze qualitative information in a systematic way [28,29] to delve deeply into the relevant OSH issues and the deficiencies of OSH management. According to the interview results, the experts’ opinions were analyzed and concluded. Based on the qualitative interview with 15 labor inspectors and 25 business executives regarding the problems of OSH in the manufacturing industries in Taiwan, the results reveal that the most important problems with OSH management are employers’ negligence and workers’ insufficient knowledge about OSH management. The biggest problems faced by medium-sized manufacturing industries in promoting OSH are the lack of support from employers, and the lack of knowledge about OSH among workers themselves. These results correspond to Oliveira’s and Nunhes et al.’s research revealing that some industries ignored OSH management, thereby causing occupational disasters [9,10].

In addition, these experts think that in order to prevent fires, organizations should advocate for the hot work permits before starting hot work, such as burning, welding, etc. In order to effectively stop such accidents, enterprises and authorities should continue promoting OSH self-inspection. The amounts of the penalties for some incidents, such as fires, should be increased. As for the storage facilities, if a company has a shortage of storage facilities for the dangerous materials, such materials can be stored in partitioned area in certain safety facilities. Additionally, in order to enhance employers’ and workers’ OSH management and knowledge, OSH education/training plans should also be strengthened. A strict review system should be adopted for training institutions. In addition, the prevention of accidents caused by repairing roofs during annual typhoon season should be advocated. Moreover, OSH education should be started in elementary and junior high schools. The chemistry curriculum content should include hazards, harmful substances, and graphic identification and describe how to use protective equipment. As for cooperative education in schools and companies, authorities should audit the contracts, which include how to train students’ attention to OSH in their workplaces. All of the qualitative results reveal the importance of creating a safe workplace to decrease the risk of employee injuries. In particular, Johnson’s research reveals that the most important facet of OSH management for an employee is creating a suitably safe workplace, one that allows employees to avoid hazards that are likely to cause death or serious harm.

The experts also provided recommendations for implementing and revising the OSH Act, which may help government organizations, employers, and workers to collaboratively participate in OSH management and hence reduce work-related injuries, incidents, diseases, or deaths [33,34]. These experts recommended that the head of OSH could also serve as the head of the management department and set up an OSH management unit, thereby contributing to OSH management. To maintain the quality of education/training, an audit system for OSH education/training implementation effects should be established, and lecturers’ qualifications and workers’ learning outcomes should be reviewed. Additionally, enterprises and authorities should pay more attention to the promotion in OSH management, especially for self-inspections, the implementation of work permission systems, and the evaluation of hazards. The records of the operating environment should be fully understood in order to make good OSH improvement measures. In addition, workers should use protective equipment to prevent dangerous accidents. While engaged in repairing roofs, workers should use safety belts or other measures to prevent falling. They should also get engaged in self-inspections and cultivate their professional abilities and their recognition of disaster-prevention measures. For instance, protective measures for mechanical devices should not be disassembled or switched. Most importantly, OSH personnel should have engineering backgrounds, and all workers should have their own training manuals, hence enabling them to keep their own OSH records before retirement.

In addition, because OSH management is quite a complex system involving multiple disciplines and OSH technical services, such as design and safety analysis, to support routine operation and installations [35], as Rasmussen [36] suggested, the risk management regarding OSH should be controlled in each specific hazard category and be investigated via a system-oriented approach with functional abstraction to investigate. Komljenovic, Loiselle, and Kumral [37] also suggested that a more systemic model taking organizational risks will be considered at the next level of OSH management. These recommendations can serve as guidelines for drafting the OSH training manuals. Through these manuals, while improving productivity, employers can also protect their workers from occupational hazards and risks [33,38].

## 5. Conclusions

Occupational diseases, injuries, and fatalities not only cause the loss of valuable workers but also lead to high healthcare expenses. Because occupational accidents and diseases can be prevented through a well-established occupational safety and health management system, in order to reduce the waste in human resources and medical and health care resources, the study investigated the shortage of work-related occupational safety and health (OSH) measures in medium-sized manufacturing industries. The qualitative research results revealed significant shortcomings of the present OSH management and process. To address these shortcomings, the results also provided recommendations for how to implement and revise the OSH Act and how to research and enhance OSH management. The results of this study will not only supply the Ministry of Labor (Taiwan) with data to plan the strategy of OSH management but also will allow employers and workers to improve OSH management in the workplace in order to prevent the occurrence of occupational accidents.

## Data Availability

Not applicable.
